# Exercise training performed simultaneously to a high-fat diet reduces the degree of insulin resistance and improves adipoR1-2/APPL1 protein levels in mice

**DOI:** 10.1186/1476-511X-11-134

**Published:** 2012-10-10

**Authors:** JM Farias, RM Maggi, CB Tromm, LA Silva, TF Luciano, SO Marques, FS Lira, CT de Souza, RA Pinho

**Affiliations:** 1 Laboratory of Exercise Biochemistry and Physiology, Postgraduate Program in Health Sciences, Health Sciences Unit, Universidade do Extremo Sul Catarinense, Criciúma, SC, 88806000, Brazil

**Keywords:** Exercise, Obesity, High-fat diet, Insulin resistance

## Abstract

**Background:**

The aim of the present study was to evaluate the protective effect of concurrent exercise in the degree of the insulin resistance in mice fed with a high-fat diet, and assess adiponectin receptors (ADIPOR1 and ADIPOR2) and endosomal adaptor protein APPL1 in different tissues.

**Methods:**

Twenty-four mice were randomized into four groups (n = 6): chow standard diet and sedentary (C); chow standard diet and simultaneous exercise training (C-T); fed on a high-fat diet and sedentary (DIO); and fed on a high-fat diet and simultaneous exercise training (DIO-T). Simultaneously to starting high-fat diet feeding, the mice were submitted to a swimming exercise training protocol (2 x 30 minutes, with 5 minutes of interval/day), five days per week, for twelve weeks (90 days). Animals were then euthanized 48 hours after the last exercise training session, and adipose, liver, and skeletal muscle tissue were extracted for an immunoblotting analysis.

**Results:**

IR, IRs, and Akt phosphorylation decreased in the DIO group in the three analyzed tissues. In addition, the DIO group exhibited ADIPOR1 (skeletal muscle and adipose tissue), ADIPOR2 (liver), and APPL1 reduced when compared with the C group. However, it was reverted when exercise training was simultaneously performed. In parallel, ADIPOR1 and 2 and APPL1 protein levels significantly increase in exercised mice.

**Conclusions:**

Our findings demonstrate that exercise training performed concomitantly to a high-fat diet reduces the degree of insulin resistance and improves adipoR1-2/APPL1 protein levels in the hepatic, adipose, and skeletal muscle tissue.

## Introduction

Obesity has been linked to genetic factors, inadequate level of physical activity, and dietary aspects such as food availability, among others [1]. Overweight and obesity result from a complex interaction between genetic, metabolic, behavioral, and environmental factors. High-calorie intakes, lowered energy expenditures, or a combination thereof lead to a positive energy balance and marked weight gain [2]. From the clinical standpoint, *obesity* is defined as a state of high body weight, more specifically of adipose tissue, that is intense enough to have adverse consequences on health [3], among which is diabetes mellitus type 2 (DM2). The link between obesity and DM2 is insulin resistance. Nevertheless, the relationship between obesity and insulin resistance is a result of changes in the insulin signal transduction pathway, with a decrease in kinase activity of insulin receptor (IR), insulin receptor substrate 1 and 2 (IRS1, IRS2), and phosphatidylinositol 3-kinase (PI3K), or both.

Insulin receptor is a protein with endogenous tyrosine kinase activity that, after activation by insulin, undergoes rapid autophosphorylation and subsequently phosphorylates intracellular protein substrates, including IRS1 and 2 [4]. IRS proteins act as messenger molecule-activated receptors to signaling with Src homology 2 domains, which are important steps in insulin action. After stimulation by insulin, IRS1and 2 associate with several proteins, including PI3K [5-7]. Downstream to PI3K, the serine/threonine kinase, Akt, is activated and plays a pivotal role in the regulation of various biological processes, including apoptosis, proliferation, differentiation, and intermediary metabolism [8,9].However, live substances such as adiponectin [10-12] serve as well-documented insulin sensitizers.

Adiponectin, an adipokine secreted by the white adipose tissue, plays an important role in regulating glucose and lipid metabolism and controlling energy homeostasis in insulin-sensitive tissues for review see [13]. Adiponectin exerts its effects through two membrane receptors, AdipoR1 and AdipoR2. Endosomal adaptor protein (APPL1) is the first identified protein that interacts directly with its receptors. The PTB domain of APPL1 interacts directly with the intracellular region of adiponectin receptors [14]. Through this interaction, APPL1 mediates adiponectin signaling and its effects on metabolism. APPL1 also functions in the insulin-signaling pathway and is an important mediator of adiponectin-dependent insulin sensitivity in skeletal muscle, adipose tissue, liver, and other organs [14]. Hence, APPL1 plays a critical role in the cross-talk between adiponectin- and insulin signaling pathways.

Acute or chronic exercise has been showed to induce numerous metabolic and hemodynamic factors that can contribute to the improvements in glucose homeostasis in individuals with insulin resistance [15-18]. These adaptive responses include enhanced insulin action on the skeletal muscle glucose transport system, reduced hormonal stimulation of hepatic glucose production, improved blood flow to skeletal muscle, and normalization of an abnormal blood lipid profile. In fact, the beneficial effects of an acute bout of exercise and of chronic exercise training on insulin action in insulin-resistant states are well established. Our group and other groups have demonstrated that the accomplishment of exercise (chronic and acute) improves insulin resistance; therefore, exercise protocols are performed when the diet-induced obesity (reversal treatment manner) is always installed in mice and rats [19-24]. In addition, Marinho and colleagues [25] have shown the involvement of APPL1 in the improvement of insulin resistance in the liver of exercise training mice. Few are knowledgeable about the efficacy of exercise training on insulin signaling/insulin resistance when applied simultaneously to the onset of a high-fat diet. Thus, the aim of this study was to investigate whether the exercise training performed simultaneously, reduce the degree of high-fat diet-induced insulin resistant and whether it was related to adiponectin receptors and APPL1 protein levels in the adipose tissue, skeletal muscle, and liver of mice.

## Methods

### Animals

Twenty-four 2-month-old male Swiss mice from the colony maintained by the Universidade do Extremo Sul Catarinense [UNESC], Criciúma, Santa Catarina, Brazil, were used in this study. The animals were randomized into four groups (n = 6): (i) control – chow standard diet and sedentary [C]; (ii) control – chow standard diet and concomitant exercise training (C-T); (iii) mice fed on a high-fat diet and sedentary (DIO); and (iv) mice fed on a high-fat diet and concomitant exercise training (DIO-T). The environment was maintained at 70% relative humidity and 20 ± 2°C and under a 12-h dark:light cycle. All procedures were conducted in accordance with regulation nº. 11794/08 (DOU 196, Section 1, October 2008) and approved by the local ethics committee.

### Diet and exercise protocol

The composition of the experimental diet was according to De Souza and colleagues [26] and was purchased from Nuvital Nutrientes SA, Brazil (Table 1). All animals had free uninterrupted access to water and food for 90 days. Simultaneously to starting high-fat diet feeding, the mice were submitted to swimming exercise training (2 x 30 minutes, with 5 minutes of interval/day), five days per week, for twelve weeks (1 week adaptation and 11 weeks exercise training periods). The mice were adapted to swimming for 20 min on the first day; 30 min on the second day, until they reached 60 minutes daily (1 week). Exercise training was conducted in a 120 × 60 × 50 cm swimming pool with 10 × 15 × 50 cm lanes and with temperature controlled at 30 ± 2°C.

**Table 1:** Components of high-fat diet and standard diet

	**Standard diet**		**High-fat diet (DIO)**	
**Ingredients**	**g.kg**^**-1**^	**kcal.kg**^**-1**^	**g.kg**^**-1**^	**kcal. kg**^**-1**^
Cornstarch (Q.S.P.)	398	1590	116	462
Casein	200	800	200	800
Sucrose	100	400	100	400
Dextrinated starch	132	528	132	528
Lard	-	-	312	2808
Soybean Oil	70	630	40	360
Cellulose	50	-	50	-
Mineral mix	35	-	35	-
Vitamin mix	10	-	10	-
_L_-Cystine	3	-	3	-
Choline	2.5	-	2.5	-
Total	1000	3948	1000	5358

### Euthanasia

Mice were anesthetized with an intraperitoneal (i.p) injection of ketamine chlorohydrate (50 mg/kg; Ketalar; Parke-Davis, Ann Arbor, MI) and xylazine (20 mg/kg; Rompun; Bayer, Leverkusen), and adipose, hepatic, and muscle tissue was extracted. In all experiments, the appropriateness of anesthesia depth was tested by evaluating pedal and corneal reflexes, throughout the experimental procedure. Following the experimental procedures, the mice were killed under anesthesia (thiopental 200 mg/kg), following the recommendations of the NIH publication.

### Protein analysis by immunoblotting

As soon as anesthesia was assured by the loss of pedal and corneal reflexes, the abdominal cavity was opened, the cava vein was exposed, and 0.2 mL of normal saline (−) or insulin ((+) 10^-6^ M) were injected (6 mice per group). After insulin injection (only for insulin pathway analysis), adipose, liver, and muscle fragments were excised. The tissues were homogenized immediately in 1.0 mL of extraction buffer (1% Triton-X 100, 100 mM Tris, pH 7.4, containing 100 mM sodium pyrophosphate, 100 mM sodium fluoride, 10 mM EDTA, 10 mM sodium vanadate, 2 mM PMSF, and 0.1 mg of aprotinin/ml) at 4°C with a Polytron MR 2100 (Kinematica, Switzerland). The extracts were centrifuged at 11000 rpm and 4°C in an eppendorf centrifuge 5804R (Eppendorf AG, Hamburg, Germany) for 40 min to remove insoluble material, and the supernatants of this tissue were used for protein quantification, according to the Bradford method. Proteins were denaturated by boiling in Laemmli sample buffer containing 100 mM DTT. Next, 0.2 mg of protein extracts obtained from each sample were separated by SDS-PAGE gel and transferred to nitrocellulose membranes. Membranes were blocked, probed, and developed. Antibodies used for immunoblotting were anti-IR, anti-phospho IR, anti-IRS1 and anti-phospho IRS1 antibodies (Cell Signaling Technology, Beverly, MA, USA), and anti-Akt, anti-phospo Akt^ser473^, anti-APPL1, ADIPOR1 and 2 antibodies (Santa Cruz Biotecnology, Santa Cruz, CA, USA). Chemiluminescent detection was performed with horseradish peroxidase-conjugate secondary antibodies (Thermo Scientific, Rockford, IL, USA). Autoradiographs of membranes were taken for the visualization of protein bands. The results of blots are presented as direct comparisons of the area of the apparent bands in autoradiographs and quantified by densitometry using the Scion Image software (Scion Image software, ScionCorp, Frederick, MD).

### Statistical analysis

All numeric results are expressed as the means ± standard error of mean (SEM) of densitometric units. The results of blots are presented as direct comparisons of bands or spots in autoradiographs and quantified by optical densitometry. Statistical analysis was performed using the ANOVA test with the Tukey post test. Significance level was established as p < 0.05. Data were analyzed using the Statistical Package for the Social Sciences (SPSS) version 18.0 for Windows.

## Results

### Physiological and metabolic parameter

The results show that DIO and DIO-T mice exhibited a significant weight gain when compared with control groups (Table 2). The DIO group presented a weight increased of 14.86 g; whereas the DIO-T group increased by 6.45 g, demonstrating that simultaneous exercise training reduces body weight gain.

**Table 2:** Body weight

**Groups**	**Body weight (mg)**
**C**	40.43 ± 1.08
**C-T**	37.14 ± 0.90
**DIO**	55.29 ± 2.00#
**DIO-T**	46.88 ± 1.83$

The results are presented as means ± SEM of n = 8, #p < 0.05 different from DIO versus control groups, and $p < 0.05 different from DIO-T versus DIO groups.

### Exercise training concomitant to high-fat diet feeding reduces degree of insulin resistance and improves adipoR1/APPL1 protein levels in the adipose tissue

**Figure 1:**
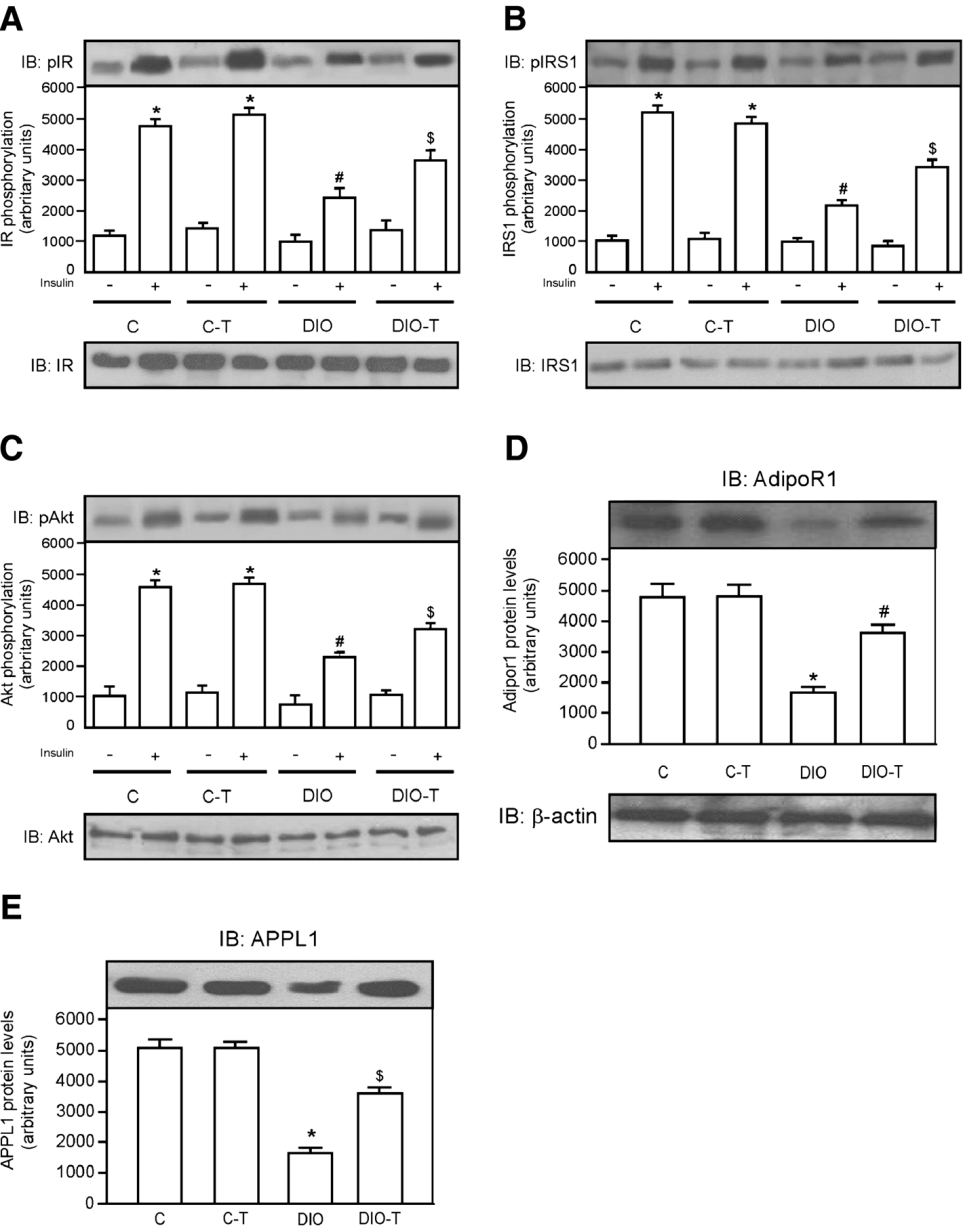
**Effects of exercise training concomitant to high-fat diet feeding on IR, IRS1, and Akt phosphorylation and adipoR1/APPL1 protein levels in the adipose tissue.** Analysis of insulin-induced tyrosine phosphorylation of IR (**A**), IRS1 (**B**), phosphorylation of Akt^Ser473^ (**C**) and ADIPOR1 (**D**) and APPL1 (**E**) protein levels. In lower panels, original membrane stripped and reblotted for IR, IRS1,and Akt total protein levels (**A**, **B** and **C**, respectively) and β-actin (**D** and **E**). The results are presented as means ± SEM of n = 6, *p < 0.05, different from C-T and C groups with insulin injection versus C-T and C groups with saline injection, ^#^p < 0.05 different from DIO versus control groups, and ^$^p < 0.05 different from DIO-T versus DIO groups.

The effects of exercise training on levels of phosphorylation of IR, IRS1, and Akt were examined in the adipose tissue of C, C-T, DIO, and DIO-T groups submitted to the exercise training. As expected, an insulin (+) injection showed increased IR, IRS, and Akt phosphorylation (Figures 1A, 1B, and 1C, respectively) in the adipose tissue of mice that were fed chow standard (sedentary and training groups) when compared with those that were administered a saline (−) injection. However, when mice were fed with a high-fat diet and not submitted to training protocol (DIO group), IR, IRS, and Akt phosphorylation (Figures 1A, 1B, and 1C, respectively) in adipose tissue were reduced, when compared with control group. On the other hand, IR, IRS, and Akt phosphorylation (Figure 1A, B, and C, respectively) in the adipose tissue of the DIO + T group increased, when compared with the DIO sedentary group. In parallel, the adiponectin receptor 1 (ADIPOR1) and its molecule adaptor APPL1 exhibit market levels reduced when compared with control sedentary and training groups (Figure 1D and 1E, respectively). However, when exercise training was performed simultaneously to high-fat diet feeding, it was partially reverted (Figure 1D and E, respectively).

### Exercise training concomitant to high-fat diet feeding reduces degree of insulin resistance and improves adipoR2/APPL1 protein levels in the hepatic tissue

**Figure 2:**
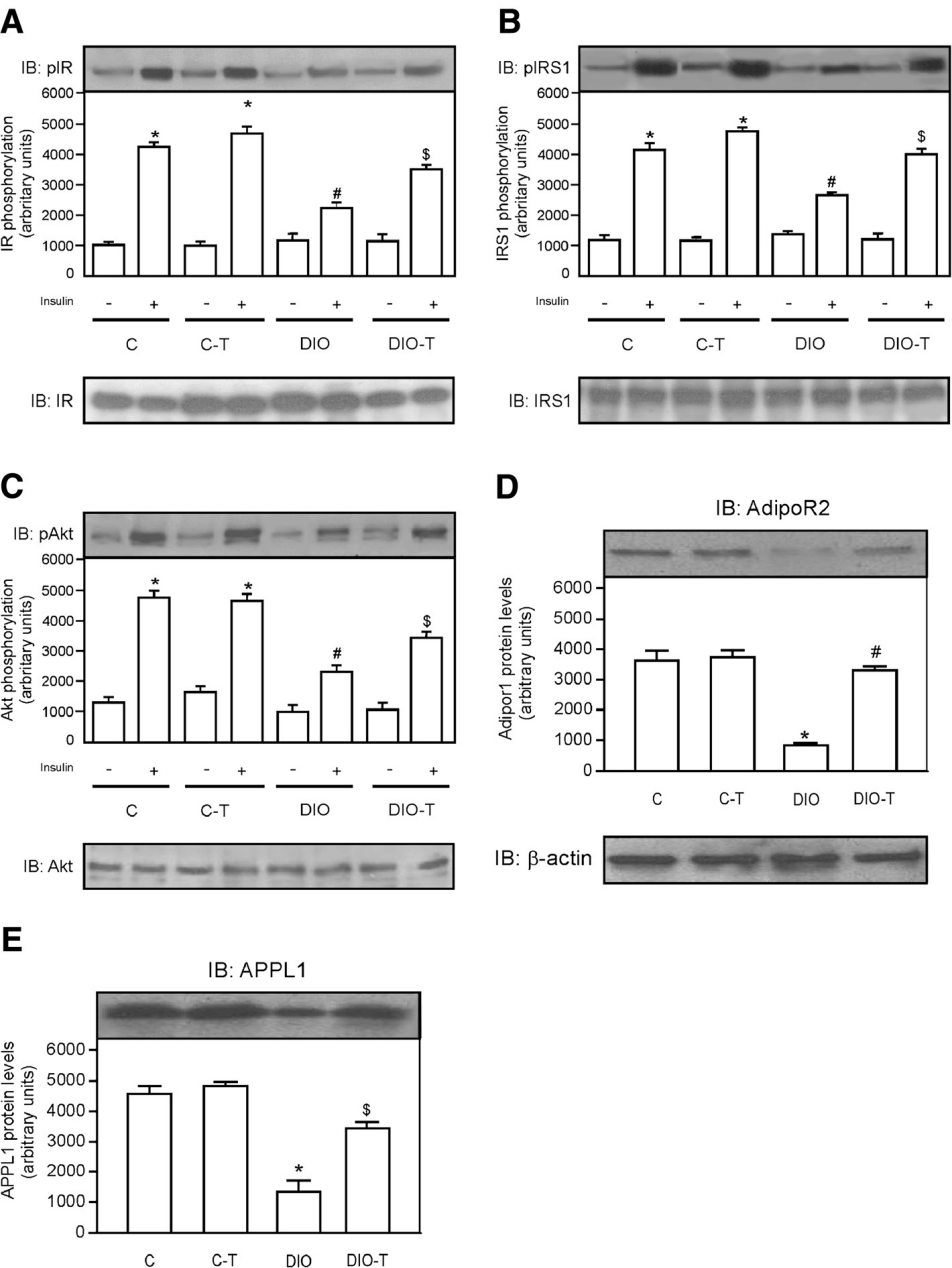
**Effects of exercise training concomitant to high-fat diet feeding on IR, IRS1, and Akt phosphorylation and adipoR2/APPL1 protein levels in the hepatic tissue.** Analysis of insulin-induced tyrosine phosphorylation of IR (**A**), IRS1 (**B**), phosphorylation of Akt^Ser473^ (**C**) and ADIPOR2 (**D**) and APPL1 (**E**) protein levels. In lower panels, original membrane stripped and reblotted for IR, IRS1, and Akt total protein levels (**A**, **B** and **C**, respectively) and β-actin (**D** and **E**). The results are presented as means ± SEM of n = 6, *p < 0.05, different from C-T and C groups with insulin injection versus C-T and C groups with saline injection, ^#^p < 0.05 different from DIO versus control groups, and ^$^p < 0.05 different from DIO-T versus DIO groups.

The effects of exercise training on levels of phosphorylation of IR, IRS1, and Akt were also examined in the hepatic tissue. The insulin (+) injection showed increased IR, IRS, and Akt phosphorylation (Figure 2A, 2B, and 2C, respectively) in the hepatic tissue of mice that were fed chow standard (sedentary and training groups) when compared with those that were administered a saline (−) injection. However, when mice were fed a high-fat diet and were not submitted to training protocol (DIO group), IR, IRS, and Akt phosphorylation (Figure 2A, B and C, respectively) in hepatic tissue reduced, when compared with the control group. On the other hand, IR, IRS, and Akt phosphorylation (Figure 2A, 2B, and 2C, respectively) in liver of the DIO + T group increased, when compared with the DIO sedentary group. In parallel, ADIPOR2 and APPL1 exhibit a marked reduction in levels when compared with control sedentary and training groups (Figure 2D and 2E, respectively). However, when exercise training was performed simultaneously to high-fat diet feeding, it was partially reverted (Figure 2D and 2E, respectively).

### Exercise training concomitant to high-fat diet feeding reduces degree of insulin resistance and improves adipoR1/APPL1 protein levels in the skeletal muscle

**Figure 3:**
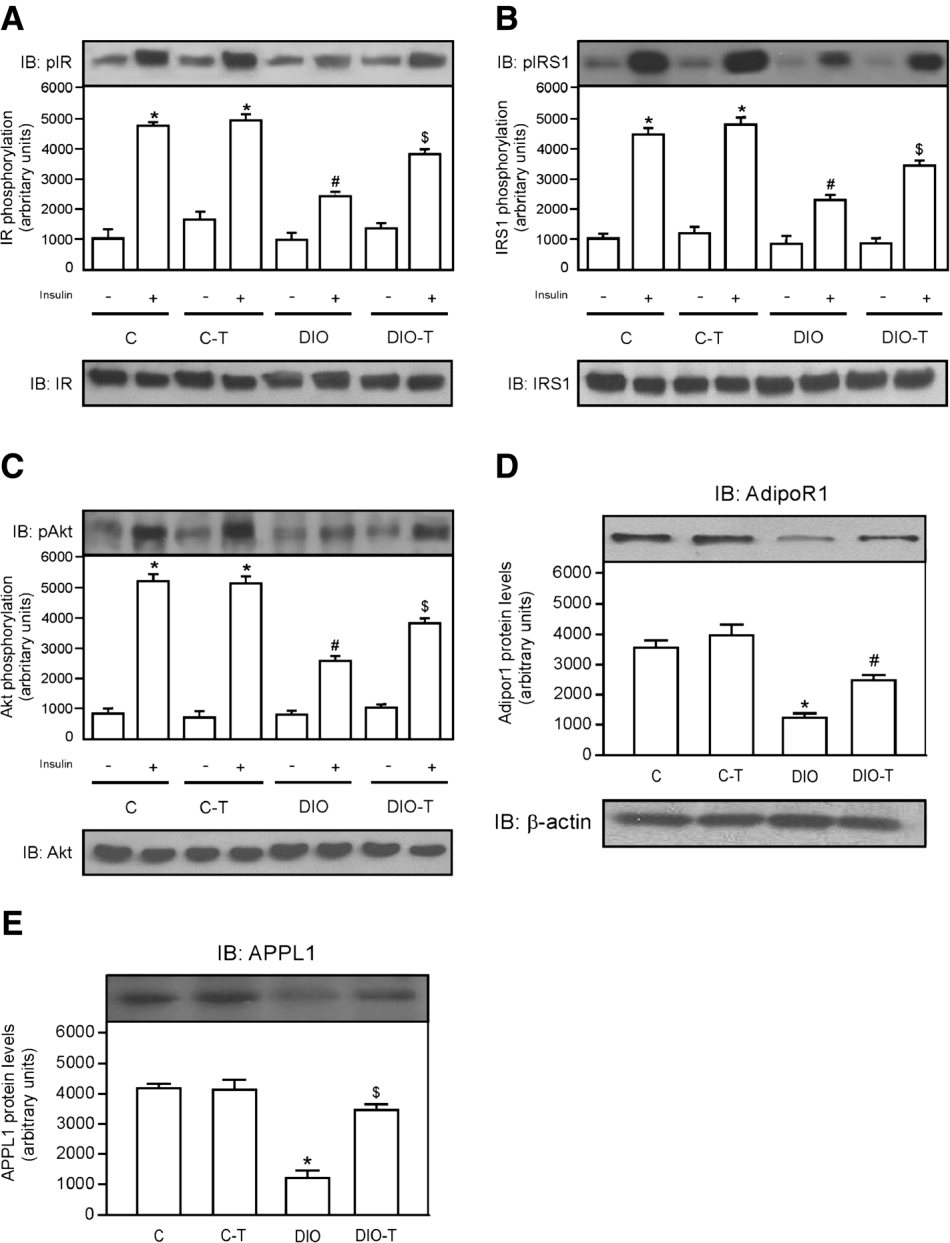
**Effects of exercise training concomitant to high-fat diet feeding on IR, IRS1, and Akt phosphorylation and adipoR2/APPL1 protein levels in the skeletal muscle*.*** Analysis of insulin-induced tyrosine phosphorylation of IR (**A**), IRS1 (**B**), phosphorylation of Akt^Ser473^ (**C**) and ADIPOR2 (**D**) and APPL1 (**E**) protein levels. In lower panels, original membrane stripped and reblotted for IR, IRS1, and Akt total protein levels (A, B, and C, respectively) and β-actin (**D** and **E**). The results are presented as means ± SEM of n = 6, *p < 0.05, different from C-T and C groups with insulin injection versus C-T and C groups with saline injection, ^#^p < 0.05 different from DIO versus control groups, and ^$^p < 0.05 different from DIO-T versus DIO groups.

As adipose and hepatic tissue, the insulin (+) injection showed increased IR, IRS, and Akt phosphorylation (Figure 3A, 3B and 3C, respectively) in the hepatic tissue of mice that were fed chow standard, when compared with those that were administered a saline (−) injection. However, when mice were fed with a high-fat diet and were not submitted to training protocol (DIO group), IR, IRS, and Akt phosphorylation (Figure 3A, 3B, and 3C, respectively) in hepatic tissue reduced, when compared with control group. On the other hand, IR, IRS, and Akt phosphorylation (Figure 3A, 3B, and 3C, respectively) in liver of the DIO + T group increased, when compared with the DIO sedentary group. In parallel, ADIPOR2 and APPL1 exhibit market reduction in levels when compared with control sedentary and training groups (Figure 3D and E, respectively). However, when exercise training was performed simultaneously to high-fat diet feeding, it was partially reverted (Figure 3D and 3E, respectively).

## Discussion

The prevalence of obesity and associated chronic diseases has increased significantly in recent years worldwide. A high-fat diet and physical inactivity have been imputed as precursors of insulin resistance, mainly in adipose, hepatic, and muscle tissue [1-3]. On the other hand, physical exercise has been described as a way of controlling glucose homeostasis and increasing insulin sensitivity in several tissues. Over the past several years, considerable progress has been made in understanding the molecular basis for these clinically important effects of physical exercise. In fact, there is now extensive epidemiological evidence demonstrating that long-term regular physical exercise can significantly reduce the body fat and insulin resistance. The molecular mechanism involved in sensitivity to insulin mediated by physical exercise is associated to the increase in levels of phosphorylation in pivotal protein related to insulin signal transduction, such as IR, IRSs, and Akt [19-24]. However, most studies have evaluated the therapeutic effects of physical exercise in individuals who already have obesity; whereas a few studies have demonstrated the impact of this intervention when applied simultaneously (protective manner) to those fed a high-fat (that wisely induces both obesity and insulin resistance). In the present study, we showed that mice which were fe with a high-fat diet and not submitted simultaneously to exercise training protocol have reduced levels of phosphorylation of IR, IRS, and Akt in hepatic, muscle, and adipose tissue, when compared with the chow standard control group. As widely expected, a high-fat diet will induce insulin resistance. On the other hand, when mice were fed with a high-fat diet and were simultaneously submitted to exercise training, the degree of insulin resistance was significantly reduced; that is, the levels of phosphorylation of IR, IRS, and Akt increased in the DIO-T group when compared with the DIO group.

Several mechanisms have been shown to link obesity and insulin resistance. Obesity has been strongly associated with a pro-inflammatory molecule (low-grade inflammation), including the IKK/NF-κB pathway [27]. Increased activation of the IKK/NF-κB pathway results in increased serine 307 phosphorylation of IRS-1 that, ultimately, leads to impaired insulin signaling in several peripheral tissue [28]. A previous study, including our group, showed that diet-induced obesity rats submitted to exercise training reduce activation of both the NF-κB and serine 307 phosphorylation of IRS-1, leading to a decreased resistance to insulin [24,27]. In addition, evidence demonstrates that exogenous nitric oxide (NO) and the NO produced by inducible nitric oxide synthase (iNOS) can induce insulin resistance by S-nitrosation [29]. So, Pauli and colleagues investigated whether this insulin resistance, mediated by S-nitrosation of proteins involved in early steps of the insulin signal transduction pathway, could be reversed by physical exercise [30]. The authors observed that physical activity can revert insulin resistance through the reduction of S-nitrosation of the IR and IRS proteins. Other well-characterized molecules that induce insulin resistance and are induced by the high-fat diet are the phosphatase protein, such as PTP, PTEN, and SOCS3. These proteins have been reported to bind to the insulin receptor and prevent the coupling of IRS-1 with the insulin receptor, thereby inhibiting IRS-1 phosphorylation and downstream insulin signaling [31,32]. Our group also showed that 12 weeks of exercise training reduced the expression of both phosphatase and SOCS3 [24].

As described earlier, a gamma of results has shown that exercise training decreases molecules activity or protein levels that can lead to insulin resistance (PTEN, PTP1B, SOCS3, pro-inflammatory molecule, S-nitrosation, and others); it can also improve insulin resistance. In addition, insulin sensitivity may be improved by increased adiponectin levels [14]. Adiponectin is adipokine that is predominantly secreted by differentiated adipocytes that are involved in energy homeostasis, insulin sensitivity, and the anti-inflammatory response [33]. The physical exercise leads to higher levels of adiponectin, and it may also reduce insulin resistance for review see [33]. Adiponectin is reduced in obesity and increased levels of this peptide by exercise training may improve insulin signal transduction [25]. Co-treatment of C2C12 myotubes with adiponectin and insulin showed a synergistic increase in Akt phosphorylation, and this synergism disappeared in APPL1 knockdown cells [14]. Hence, the involvement of adiponectin in this process is demonstrated. Here, we showed that mice which were fed with a high-fat diet and not submitted simultaneously to exercise training protocol (DIO group) have reduced adiponectin receptor R1 and 2 and APPL1 protein levels in hepatic, muscle, and adipose tissue, when compared with the chow standard control group. On the other hand, when mice were fed with a high-fat diet and simultaneously submitted to exercise training, the degree of reduction of ADIPOR1-2 and APPL1 was significantly smaller; that is, ADIPOR1-2 and APPL1 protein levels increased in the DIO-T group when compared with the DIO group.

Adiponectin exerts its action through its receptors ADIPOR1 and ADIPOR2. ADIPOR1 and ADIPOR2 interact with the adaptor protein that contains a pleckstrin homology domain, a phosphotyrosine domain, and a leucine zipper motif (APPL1), which bind the N-terminal intracellular domains of the receptors [10-13]. It has been demonstrated that the endosomal adaptor protein, APPL1, regulates the activity of Akt [14]. The improvement found that insulin signaling in different tissues studied in the present study, least in part, can be attributed to recovery protein levels in adiponectin receptors and APPL1. Previous studies have demonstrated that exercise training induces increased adiponectin levels [34,35], and ADIPOR1 protein levels in skeletal muscle [35]. In parallel, Marinho et al. [25] have found that exercise increases insulin action, at least in part, through the enhancement of APPL1 expression in the liver of obese mice. Our results confirm that an improvement in insulin action can be, least in part, via adipoR1-2/APPL1. Taken together, our data demonstrated that exercise training performed concomitantly to feeding with a high-fat diet reduces the degree of insulin resistance and improves adiponectin receptors 1 and 2 and APPL1 protein levels in the hepatic, adipose, and skeletal muscle tissue.

## Abbreviations

ADIPOR1: Adiponectin receptor 1; ADIPOR2: Adiponectin receptor 2; APPL1: Endosomal adaptor protein 1; DIO: Diet induces obesity; IR: Insulin receptor; IRS-1: Insulin receptor substrate 1; IRS-2: Insulin receptor substrate 2; PI3K: Phosphatidylinositol 3-kinase; Akt: Protein kinase B; DM2: Diabetes mellitus type 2.

## Competing interests

The authors declare that they have no competing interests.

## Authors’ contributions

JMF, RMM, CTS, and RAP designed research; KFB, LAS, TFL, and SOM conducted research; JMF, FSL, CTS, and RAP analyzed data; JMF, FSL, CTS, and RAP wrote the article. All authors read and approved the final manuscript.
